# The pH Value Control of Morphology and Luminescence Properties of Gd_2_O_2_S: Tb^3+^ Phosphors

**DOI:** 10.3390/ma15020646

**Published:** 2022-01-15

**Authors:** Peng Jiang, Zhipeng Li, Wei Lu, Yi Ma, Wenhuai Tian

**Affiliations:** School of Materials Science and Engineering, University of Science and Technology Beijing, Beijing 100083, China; g20198374@xs.ustb.edu.cn (P.J.); lvwei131163@163.com (W.L.); 18252581996@163.com (Y.M.); wenhuaitian@ustb.edu.cn (W.T.)

**Keywords:** Gd_2_O_2_S:Tb^3+^, morphology, pH, photoluminescence, cathodoluminescence

## Abstract

Developing rare-earth doped oxysulfide phosphors with diverse morphologies has significant value in many research fields such as in displays, medical diagnosis, and information storage. All of the time, phosphors with spherical morphology have been developed in most of the related literatures. Herein, by simply adjusting the pH values of the reaction solution, Gd_2_O_2_S:Tb^3+^ phosphors with various morphologies (sphere-like, sheet-like, cuboid-like, flat square-like, rod-like) were synthesized. The XRD patterns showed that phosphors with all morphologies are pure hexagonal phase of Gd_2_O_2_S. The atomic resolution structural analysis by transmission electron microscopy revealed the crystal growth model of the phosphors with different morphology. With the morphological change, the band gap energy of Gd_2_O_2_S:Tb^3+^ crystal changed from 3.76 eV to 4.28 eV, followed by different luminescence performance. The samples with sphere-like and cuboid-like microstructures exhibit stronger cathodoluminescence intensity than commercial product by comparison. Moreover, luminescence of Gd_2_O_2_S:Tb^3+^ phosphors have different emission performance excited by UV light radiation and an electron beam, which when excited by UV light is biased towards yellow, and while excited by an electron beam is biased towards cyan. This finding provides a simple but effective method to achieve rare-earth doped oxysulfide phosphors with diversified and tunable luminescence properties through morphology control.

## 1. Introduction

Lanthanide compounds have attracted much attention due to their wide applications in functional systems such as high-resolution displays, integrated optical systems, and memory devices [1,2,3]. As one of the most commercially successful lanthanide compound phosphors, close attention has been paid to oxysulfides in both scientific research and commercial fields. Gadolinium oxysulfide (Gd_2_O_2_S) is one of the ideal hosts for rare-earth doping with for X-ray scintillator materials due to its wide bandgap (4.6~4.8 eV) [4], low phonon energy (520 cm^−1^) [5], high chemical and thermal stability [3,6], high intrinsic conversion efficiency (12–25%) [7,8]. The terbium-doped gadolinium oxysulfide (Gd_2_O_2_S:Tb^3+^) exhibits bright green luminescence and high luminescence efficiency excited by ultraviolet, electron beam and X-ray [9]. Therefore, as a kind of high-performance phosphor, it is widely used in X-ray imaging, cathode ray luminescence and many other fields [10,11].

The luminescence properties of phosphors are strongly related to their chemical compositions, phase compositions, particle size and their morphologies [12,13,14,15]. Other methods of changing luminescence properties are relatively complex, and the change trend is relatively single, morphology control can change properties of various materials significantly since morphological changes can make a variety of basic properties change. For example, rod-like phosphors have better crystallization performance, while sphere-like phosphors have a narrower band gap. As a result, some high-performance lanthanide compounds with different morphologies, such as rod-like, flowers-like, and sheet-like, have been prepared [1,16,17,18,19].

In the latest several decades, some efforts have been made to synthesize Gd_2_O_2_S:Tb^3+^ with specific morphologies and particle size. Huang et al. fabricated ultrathin Gd_2_O_2_S:Tb^3+^ scintillation ceramics from the uniformly doped nanopowder [10]; Liu et al. synthesized sheet-like Gd_2_O_2_S:Tb^3+^ scintillation ceramics via hydrogen reduction [18]; Sang et al. synthesized multicolor Gd_2_O_2_S: xTb^3+^, yEu^3+^ hollow spheres through a template-free solvothermal method [20]. However, it should be noted that there is still a challenge to develop a simple and effective method using a single factor in synthesis condition to widely modulate the morphologies and luminescence properties.

The pH value tuned ion self-assembly and electrostatic force has been proved to be an effective method for supramolecular aggregates and constructing specific functional nanostructures. Wan et al. studied the influence of pH on the morphology and photocatalytic performance of BiVO_4_ [21]. In this paper, Gd_2_O_2_S:Tb^3+^ phosphors with controllable morphology, including sphere-like, sheet-like, cuboid-like, rod-like and flat square-like, were synthesized by hydrothermal method by changing the pH value of the reaction resolution, and then calcined at high temperature. The influence of the pH values of the reaction resolution on the crystal growth and formation mechanism of different morphologies were studied. Furthermore, the dependence of Gd_2_O_2_S:Tb^3+^ luminescence performance on different morphologies has been also discussed.

## 2. Materials and Methods

### 2.1. Preparation

Firstly, rare-earth nitric acid (Gd(NO_3_)_3_ and Tb(NO_3_)_3_) solutions were prepared by dissolving the Gd_2_O_3_ (from Beijing Honghu United Chemical products Co., LTD, Beijing, China) and Tb_4_O_7_ (from Shanghai Yuanye Biological Technology Co., LTD, Shanghai, China) in nitric acid (the concentration is 68%) at 45 °C. Secondly, 1.95 mL of Gd(NO_3_)_3_ (1 M) and 1 mL of Tb(NO_3_)_3_ (0.05 M) were added into 25 mL of ethylene glycol (EG) at 45 °C, the mole ratio of Gd to Tb is 19, then 2.0 g PVP K30 was dissolved fully in the above solution and stirred vigorously. Then 10 mL ethanol solution containing 0.11 g SC(NH_2_)_2_ was dropped at high temperature. Subsequently, sodium hydroxide solution (2 M) was dropped to adjust the pH value. The resulting solution was stirred for another 60 min at room temperature. Then the solution was poured into a 100 mL Teflon-lined stainless autoclave, put it in a drying oven and kept it warm for 18 h at 200 °C. After the hydrothermal reaction was completed, the precursor was centrifuged, washed with ethanol and ultrapure water for three times, and dried at 60 °C in the air-blast drying oven. The precursors were calcined at 750 °C in an inert atmosphere containing sulfur for 2 h to obtain the final phosphors. Heating rate was set as 8 °C min^−1^, and the nitrogen flow rate was 70 sccm.

### 2.2. Characterizaton

The phase and crystal structure of the precursors and phosphors were characterized by Bruker D8 X-ray powder diffractometer with Cu Kα radiation (λ = 0.15405 nm). The morphology and particle size were observed by a field emission scanning electron microscope (FE-SEM, Hitachi S-4800, Hitachi, Tokyo, Japan) equipped with an energy-dispersive spectroscopy (EDS). The absorbancy of phosphors were measured by a UV-3600 UV-vis-NIR spectrophotometer. The photoluminescence (PL) spectra were measured by an Edinburgh Instruments FLSP 920 fluorescence spectrophotometer equipped with a 450 W Xenon lamp (source atmosphere: air, excitation-slit/detection-slit: 7 nm/7 nm, integral time: 0.2 s, step width: 1.0 nm), the prepared sample weight is 0.15 g. Luminescence lifetime was measured in the same spectrophotometer using a nanosecond flashlamp as the excitation source. Quantum yields were measured by a C11347–11 Quantaurus-QY measurement system. Cathodoluminescence (CL) spectra were measured by an Gatan MonoCL4/Goldenscope Rainbow SEM-CL. The Commission Internationale de L’Eclairage 1931 chromaticity (CIE) coordinates were calculated from the spectra based on the 1931 CIE standard for colorimetry.

## 3. Results and Discussion

### 3.1. Morphology 

**Figure 1:**
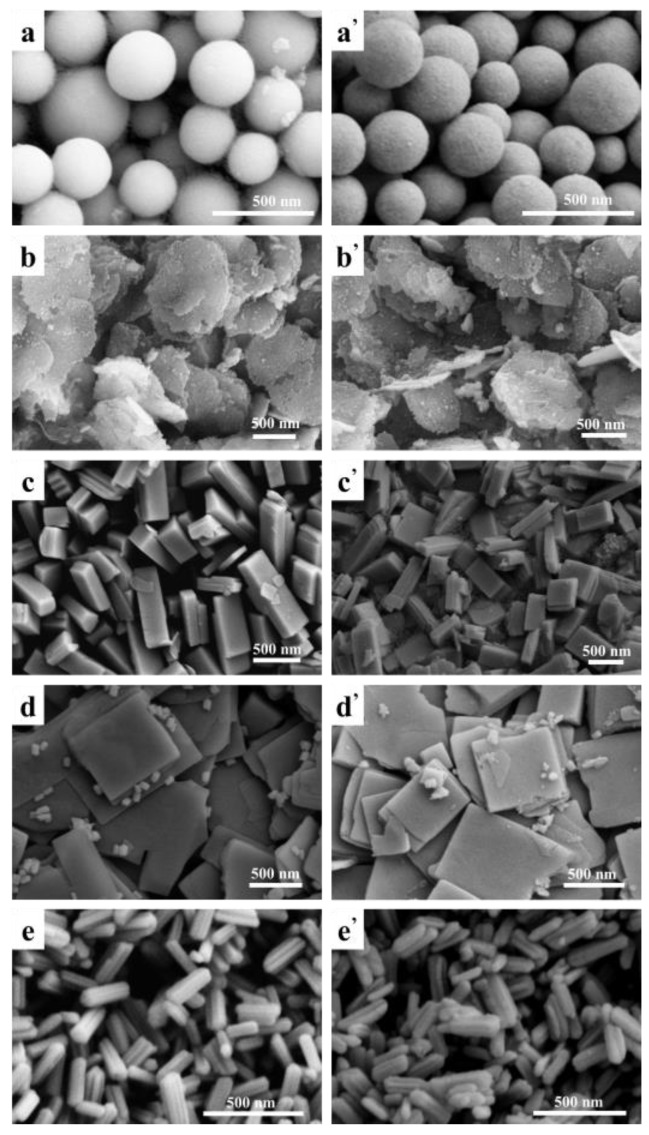
SEM images of the uncalcined Gd_2_O_2_S:Tb^3+^ precursors synthesized with different pH value of the reaction system, before and after annealing. The pH value of 5.0 (**a**,**a**’), 7.0 (**b**,**b**’), 8.0 (**c**,**c**’), 10.5 (**d**,**d**’), and 13 (**e**,**e**’).

Figure 1 shows the morphologies of the uncalcined precursors (with different pH value of the reaction system) and annealed phosphors with different pH value of the reaction system: (a) pH = 5; (b) pH = 7; (c) pH = 8; (d) pH = 10.5; (e) pH = 13. As shown in Figure 1a, the uncalcined precursor consists of well-separated spheres with diameter of 250–350 nm when pH value is 5, while the precursors synthesized with pH value of 7 show a sheeted structure with overlap and interweave seen in Figure 1b. Figure 1c indicated that the precursors obtained at pH value of 8 composed of cuboid, and when the pH value was moderately increased to 10.5, the cuboid became flatter and rod-like precursors was beginning to emerge. In the end, with increasing the pH value to 13.0, the precursors entirely consist of uniform rod-like in high yield with lengths of 200–300 nm and diameters of about 50 nm, as shown in Figure 1e.

After being annealed at 700 °C, the obtained Gd_2_O_2_S:Tb^3+^ phosphors all maintained the morphologies of their corresponding precursors, as shown in Figure 1a’–e’.

**Figure 2:**
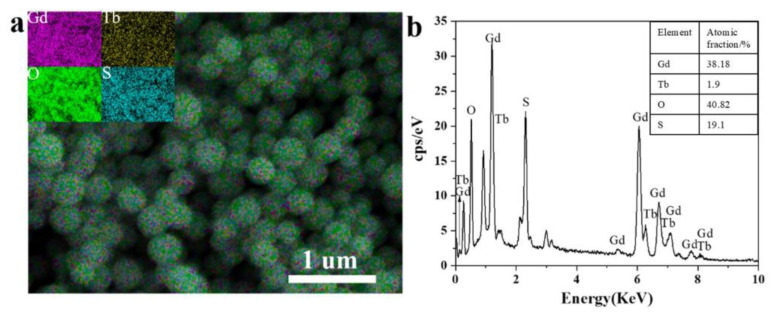
(**a**) Elements mapping and (**b**) EDS spectrum of Gd_2_O_2_S:Tb^3+^ nanospheres. Inserted is the quantification of elements content.

Elements mapping by analysis shown in Figure 2a demonstrates elements are uniformly distributed in the Gd_2_O_2_S:Tb^3+^ nanospheres after annealing. It can be seen from Figure 2b that there are gadolinium (Gd), terbium (Tb), oxygen (O) and sulfur (S) elements in Gd_2_O_2_S:Tb^3+^ phosphors and the molar ratio of (Gd + Tb)/S (2.02) is quite close to the nominal chemical stoichiometry of 2, which agrees well with the chemical formula. The results (the quantification of EDS spectrums) of samples with different morphologies are similar.

### 3.2. Structures

**Figure 3:**
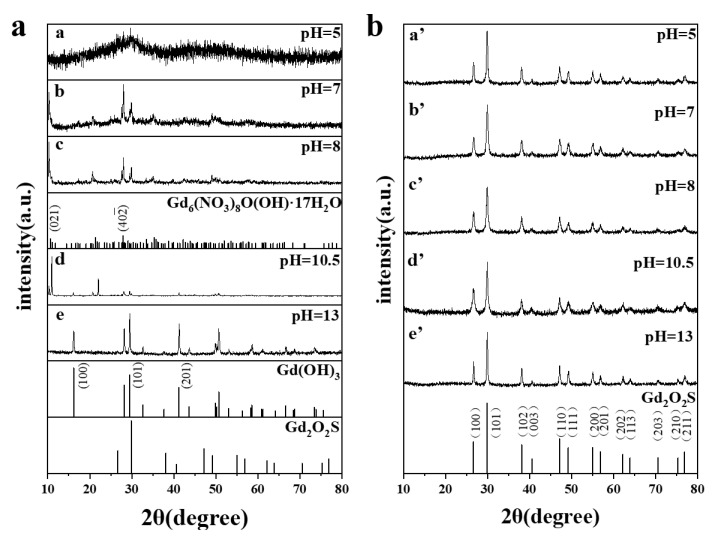
(**a**) XRD patterns of uncalcined precursors obtained at different pH values and the JCPDS cards No. 44-0437, No. 83-2037 and No.26-1422; (**b**) XRD patterns for the Gd_2_O_2_S:Tb^3+^ phosphors after annealing with different pH values.

Figure 3a shows the XRD patterns of the uncalcined precursors obtained at different pH values (with other parameters being constant) and the reference diffraction data of Gd_6_(NO_3_)_8_O(OH)_8_·17H_2_O (JCPDS No.44-0437), Gd(OH)_3_ (JCPDS No.83-2037) and Gd_2_O_2_S (JCPDS No.26-1422), respectively. When the pH value is 5.0, There is only a broad peak near 2θ = 29.7 in the curve of the precursor, indicating that the precursor is amorphous and has a very low degree of crystallinity. Their major diffraction peaks both can be indexed to monoclinic phase of Gd_6_(NO_3_)_8_O(OH)_8_·17H_2_O (JCPDS No.44-0437) when the precursors that obtained at pH = 7.0 and 8.0, except some other little diffraction peaks. So, the precursors obtained at pH = 7.0 and pH = 8.0 are inferred to have a structural of Gd_6_(NO_3_)_8_O(OH)_8_·17H_2_O. When the pH value was increased to 10.5, some new diffraction peaks emerged which can be indexed to Gd(OH)_3_ (JCPDS No.83-2037). All diffraction peaks of the precursor obtained at pH value of 13 can be well indexed to hexagonal phase Gd(OH)_3_ (JCPDS No.83-2037). Which indicates that the precursor forms Gd(OH)_3_ with high crystallinity when the alkalinity is strong in the reaction solution. Gd_2_O_2_S (JCPDS No.26-1422) do not appear in the XRD of any precursor. Figure 3b shows XRD patterns for the Gd_2_O_2_S:Tb^3+^ phosphors after annealing at 750 °C. All XRD patterns are similar to each other and they can be precisely indexed to the pure hexagonal phase of Gd_2_O_2_S (JCPDS No. 26-1422), with no other phases detected. Since the radius of Tb^3+^ is smaller than Gd^3+^, the lattice parameters of the sample become smaller after Tb doping into Gd_2_O_2_S lattices, therefore, diffraction peaks would shift to larger angles according to the Bragg law. As shown in Figure 3, the diffraction peaks of all samples shift to larger angles than the standard diffraction peak of Gd_2_O_2_S, suggesting the successful Tb doping.

**Figure 4:**
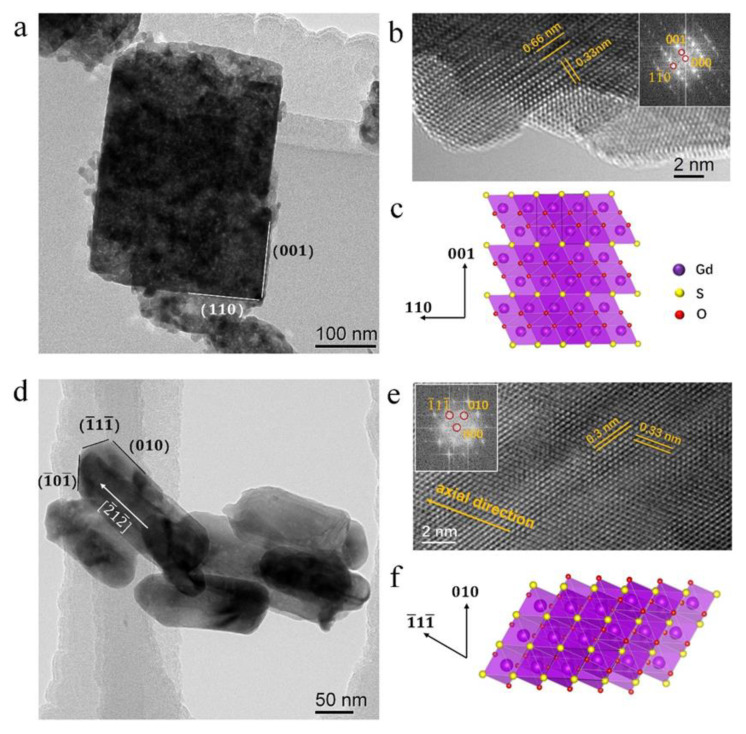
The TEM analysis of the calcined phosphor (**a**) with cuboid morphology, (**b**) the HRTEM image, the inserted is the fast Fourier transform (FFT) the image, (**c**) the corresponding crystal model. (**d**,**e**) are the TEM images of the phosphor with rod-like morphology, the inserted in e is the FFT of the HRTEM image, (**f**) the crystal model of the rod-like phosphor, the yellow axis is along the axial direction.

The TEM analysis of nanostructures with different morphology is shown in Figure 4. It could be seen that the phosphor synthesized with pH value of 8 shows a rectangular shape (384 × 292 nm^2^). The HRTEM images in Figure 4b shows the atomic structure of the sample. The crystal plane with lattice spacing of 0.66 nm and 0.33 nm could be indexed as (001) and (11$\bar{0}$) planes, respectively. The zone axis is [110] and the crystal plane which is perpendicular to the electron beam is (110). Figure 4c shows the in-plane crystal model of the synthesized phosphor with cuboid morphology. The sample in Figure 4d is the one synthesized with pH value of 13 (rod-like morphology). They are 180 nm to 220 nm along the axial direction and the diameters are ~80 nm. The lattice spacing of 0.3 nm and 0.33 nm correspond to lattice planes of ($\bar{1}$1$\bar{1}$) and (010), shown in Figure 4e. The zone axis of the image is determined as [10$\bar{1}$] and the axial direction of the rod-like phosphors is determined as [010]. Figure 4f demonstrates the crystal model of the phosphor.

The pH value of reaction solution plays a crucial role in the morphology tuning of Gd_2_O_2_S:Tb^3+^ phosphors. Apparently, the formation mechanism of the phosphors with different morphologies can be explained by the growth process of solution-recrystallization and self-assembly [12].

The formation of sphere-like morphology (when the pH value is 5) is due to the relatively high concentration of free H^+^ ions in solution. The growing particles can be adsorbed on all exposed crystal facets located at the surface of the primary nucleated nanoparticles uniformly. Finally, the isotropy grown of the primary particles results in the formation of nanospheres.

The formation of sheet-like morphology (pH = 7) due to the weaker repulsion between each primary particles and anisotropic growth due to changes in the electrostatic force in solution. the uncalcined precursors obtained at pH = 7 selective grow on specific crystal planes. It can be seen from Figure 3a that the selected crystal planes are presumed to (021) and (40$\bar{2}$) of Gd_6_(NO_3_)_8_O(OH)_8_·17H_2_O.

When the pH value is increased to 8, the concentration of OH^-^ ions is higher than that of the H^+^ ions, always the growth rate of a crystal face is determined by the relative specific energy of each face. It can be seen from Figure 3a that the uncalcined precursors obtained at pH = 8 still selective grow on (021) and (40$\bar{2}$), however, the peak of (021) plane increase and the peak of (40$\bar{2}$) plane decrease. So, the anisotropic crystal growth leads to the formation of the cuboid-like morphology.

The physicochemical properties of PVP are not affected by pH, Thiourea loses a proton to form anions and water in the basic medium, see Equation (1).
(1)
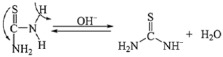


In this anion, the double bond position changes from C=S to C=N, resulting in a negative charge in the sulfur (S^−^) ion, see Equation (2) [22].
(2)
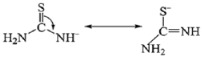


When the pH value is increased to 10.5, there exist excess OH^-^ ions in the solution. On the one hand, which is preferable for the anisotropic growth. So, the cuboid-like morphology become flatter. On the other hand, excess OH^-^ ions and Gd^3+^ leads to the formation of Gd(OH)_3_ with rod-like morphology.

With increasing pH of the reaction solution to 13, the concentration of OH^-^ ions in the reaction solution increases, resulting in increased chemical repulsion between elementary particles, forming colloidal particles. Then the rod-like seeds are formed due to the anisotropic hexagonal structure of colloidal particles. Subsequently, the continuous supplies of Gd^3+^ and OH^-^ ions to the seeds surfaces leads to the formation of the rod-like morphology [23].

**Figure 5:**
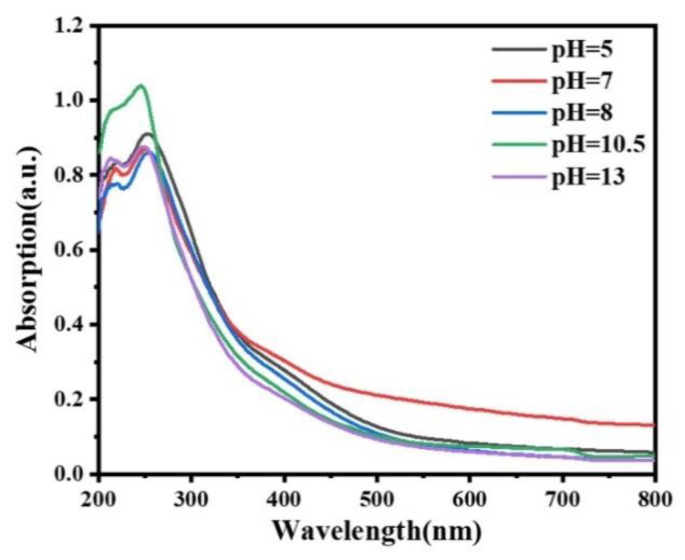
UV-vis absorption spectra of the Gd_2_O_2_S:Tb^3+^ phosphors obtained at different pH values. pH = 5.0: sphere-like, pH = 7.0: sheet-like, pH = 8.0: cuboid-like, pH = 10.5: flat square-like, pH = 13: rod-like.

To investigate the morphology dependence of bandgap, UV-vis absorption spectra were measured and shown in Figure 5. According to the results, all Gd_2_O_2_S phosphors were nearly transparent in the visible region, and had a relatively high absorbance in the wavelength range below 350 nm. The value of the optical band gap can be calculated through Tauc plot Formula, (αhv)^1/*n*^ = A(hv − *E*_g_), where α is the absorbency index, h and v are the Planck constant and frequency, A is the constant term, and *E*_g_ is the band gap width of semiconductor. As a direct semiconductor, the n value of Gd_2_O_2_S is 2. During the experiment, the ordinate of the spectrum measured by the diffuse reflectance spectrum was Abs, and it was in direct proportion to α. There is no influence on *E*_g_ value using Abs or α in Tauc plot formula. So, Abs can be used instead of α in this formula.

The optical band gaps of sphere-like, sheet-like, cuboid-like, flat square-like and rod-like phosphor was 3.89 eV, 3.99 eV, 3.93 eV, 4.05 eV, and 4.09 eV by calculating, respectively. For the samples with different morphologies, their structure sizes in three dimensional are hundreds of nanometers, which are far away from the quantum confinement regime [24]. We think the main contributing factor for little variation of band gaps may be the defects. The defects such as structural dislocations, oxygen vacancies and local variation of chemical stoichiometry during crystal growth can generate local energy states and affect optical absorption as well as the band gap measurement results [25].

### 3.3. Luminescence Properties

#### 3.3.1. Photoluminescence

**Figure 6:**
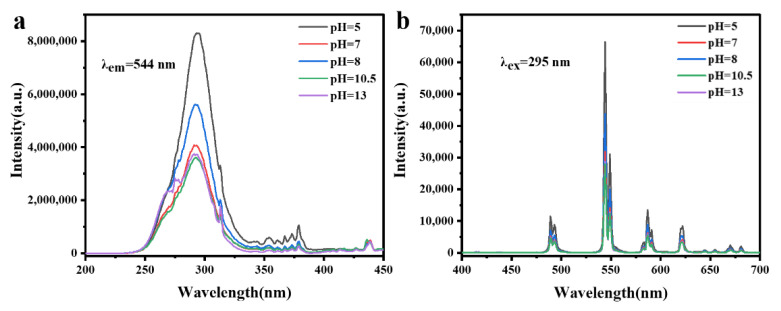
(**a**) curves of Tauc plot of the Gd_2_O_2_S:Tb^3+^ phosphors obtained at different pH values. pH = 5.0: sphere-like, pH = 7.0: sheet-like, pH = 8.0: cuboid-like, pH = 10.5: flat square-like, pH = 13: rod-like. (**b**) The optical band gap value of samples.

Figure 6 shows the photoluminescence excitation (PLE) and emission (PL) spectra of the Gd_2_O_2_S:Tb^3+^ phosphors obtained at different pH values. In the PLE spectra of Figure 6a, a strong broad-band excitation ranging from 250 to 320 nm can be observed, which is mainly aroused by the 4f^8^-4f^7^5d^1^ inter-configurational Tb^3+^ transition (λ_em_ = 544 nm). Several Gd^3+^ transitions centered at 275 nm (^8^S_7/2_ → ^6^I_J_) and 312 nm (^8^S_7/2_ → ^6^P_J_) are overlapped with the Tb^3+^ transition, and may contribute to broadening the excitation band in a certain degree [18,26]. A number of weak absorption peaks at 350–380 nm correspond to the spin-forbidden 4f-4f transition (^7^F_6_ → ^5^G_2,3_, ^7^F_6_ → ^5^L_1,5,10_, ^7^F_6_ → ^5^D_3_) [3,27]. With the change of morphology of Gd_2_O_2_S:Tb^3+^ phosphors, the intensity of excitation peak also changes. It is noted that the sphere-like and cuboid-like showed a strong excitation while sheet-like microstructures exhibited a weaker excitation intensity. Further, the excitation intensity for the flat square-like and rod-like are similar but weaker.

In the PL spectra of Figure 6b, the all obtained emission spectrum of different morphologies exhibited four obvious peaks centered at 489, 544, 587, and 621 nm, that can ascribe to the transitions from the ^5^D_4_ excited-state to the ^7^F_J_ (J = 6, 5, 4, 3) ground states of the Tb^3+^ ions, respectively. It can be found that the sphere-like microstructures exhibit the strongest PL intensity, and then cuboid-like, sheet-like, rod-like and flat square-like. The PL intensity of different morphologies can be explained by the band gap energy. Combined with Figure 5 and Figure 6, It can be seen that the smaller the band gap energy is, the higher the luminous intensity is. Apparently, smaller band gap energy is more favorable for electron transition, and thus increases the PL intensity of phosphors. Quantum yields of phosphors were also measured, the obtained quantum yields are 15.9%, 6.6%, 8.7%, 5.8%, 5.6% for pH = 5, 7, 8, 10.5, and 13, respectively. The trend of quantum yield with change of pH values is similar as the trend of PL spectra intensities.

#### 3.3.2. Cathodoluminescence

**Figure 7:**
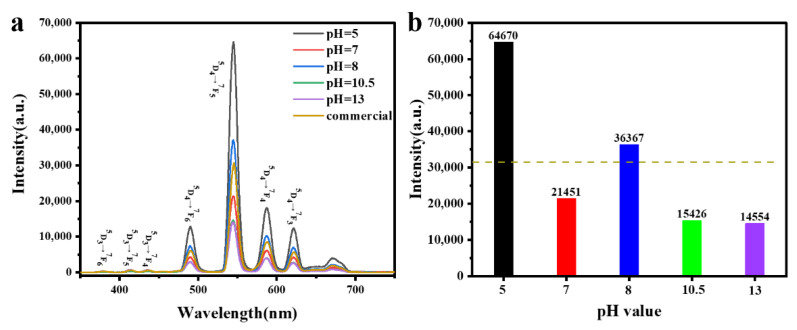
(**a**) The CL spectra of commercial cathode ray Gd_2_O_2_S:Tb^3+^ phosphors and phosphors obtained at different pH values. pH = 5.0: sphere-like, pH = 7.0: sheet-like, pH = 8.0: cuboid-like, pH = 10.5: flat square-like, pH = 13: rod-like. (**b**) The CL intensity of commercial cathode ray Gd_2_O_2_S:Tb^3+^ phosphors (dotted line) and phosphors obtained at different pH values.

Figure 7a shows the cathodoluminescence (CL) emission performances of Gd_2_O_2_S:Tb^3+^ phosphors obtained at different pH values under an accelerating voltage of 20 kV. the primary emission peaks centered at 489, 544, 587, and 621 nm corresponding to the transition from ^5^D_4_ excited-state to the ^7^F_J_ (J = 6, 5, 4, 3) ground states of Tb^3+^ ions, respectively. Location and intensity sequence of the four primary emission peaks of different morphologies are similar as the PL emission. In addition, the other peaks are ascribed to ^5^D_3_ → ^7^F_6_ (379 nm), ^5^D_3_ → ^7^F_6_ (413 nm), ^5^D_3_ → ^7^F_6_ (436 nm) transitions of Tb^3+^ ions, respectively. The spectral energy distribution is closely related to the concentration of Tb^3+^ ions, the ^5^D_3_ emission peak weakened and the ^5^D_4_ emission peak enhanced with increasing concentration of Tb^3+^ ions. So in order to decrease violet-blue emission, more activators can be doped with Gd_2_O_2_S:Tb^3+^ phosphors excited by electron beam. The order of CL intensity is the same with PL for the Gd_2_O_2_S:Tb^3+^ phosphors with different morphologies. In addition, The CL intensity of commercial cathode ray Gd_2_O_2_S:Tb^3+^ phosphors and phosphors obtained at different pH values were comparatively investigated in Figure 7b. It can be found that the sphere-like and cuboid-like microstructures exhibit the stronger CL intensities than commercial Gd_2_O_2_S:Tb^3+^ phosphors product (with average particle size of 2.8 μm). The intensity of luminescence depends on the crystallinity, density of defects and so on, therefore, our synthesized samples with size of nanometer scale may have much better crystallinity, allowing the fabrication of display screen with very high resolution.

#### 3.3.3. CIE chromaticity Diagram

**Figure 8:**
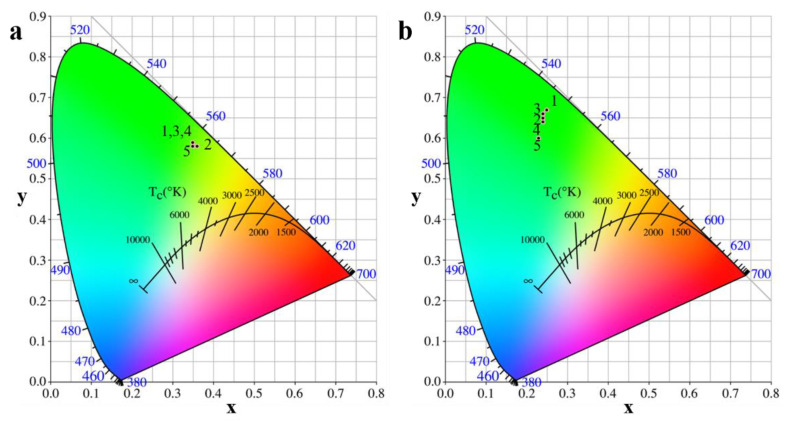
CIE chromaticity diagram of Gd_2_O_2_S:Tb^3+^ phosphors obtained (**1**) pH = 5.0: sphere-like, (**2**) pH = 7.0: sheet-like, (**3**) pH = 8.0: cuboid-like, (**4**) pH = 10.5: flat square-like, (**5**) pH = 13: rod-like at different pH values excited by (**a**) 295 nm UV light, (**b**) excited by cathode ray.

In order to visually show the color emission along with the changes for morphologies of Gd_2_O_2_S:Tb^3+^ phosphors, the CIE chromaticity diagram, excited by 295 nm UV light radiation, is presented in Figure 8a. the CIE chromaticity coordinates were discovered to be a (0.35, 0.59), b (0.36, 0.58), c (0.35, 0.59), d (0.35, 0.59), and e (0.35, 0.58). It can be seen that the sphere-like, cuboid-like and flat square-like had the same color emission of pure green while rod-like microstructures exhibited a yellow green. Obviously, the sheet-like microstructures exhibited a more yellow emission.

Figure 8b represents the CIE coordinates for Gd_2_O_2_S:Tb^3+^ phosphors of different morphologies excited by an electron beam. It can be found that color tunable blue green to pure green emission can be obtained with the changes of morphologies from rod-like to sphere-like. Specifically, the CIE coordinates for the Gd_2_O_2_S:Tb^3+^ phosphors could be changed from the e (x = 0.23, y = 0.60) to a (x = 0.25, y = 0.67). This color transition sequence was same to the increase of intensity of emission CL peak. It needs to be emphasized that color coordinates and luminescent intensity can only be explained by the effect of traps (i.e., Tb^3+^ ions migrate through a large number of Tb^3+^ ions before being emitted). However, even for the purest crystals, these centers can relax to their ground state by multiphoton emission, and inhibit the luminescence [28]. On the whole, according to the emission spectrum of different excitation sources, the color emission of Gd_2_O_2_S:Tb^3+^ phosphors excited by UV light was biased towards yellow, while excited by an electron beam was biased towards cyan due to ^5^D_3_ level of Tb^3+^.

#### 3.3.4. Luminescence Decay Curves

**Figure 9:**
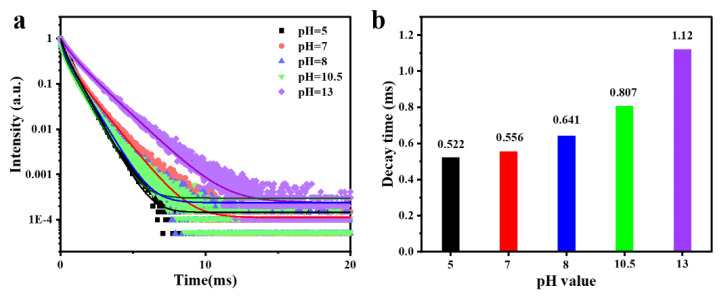
(**a**) Luminescence decay curves and (**b**) the decay time of the Gd_2_O_2_S:Tb^3+^ phosphors obtained at different pH values. pH = 5.0: sphere-like, pH = 7.0: sheet-like, pH = 8.0: cuboid-like, pH = 10.5: flat square-like, pH = 13: rod-like.

Figure 9 shows the luminescence decay curves of Gd_2_O_2_S:Tb^3+^ phosphors obtained at different pH values. It can be found that all decay curves can be fitted with a multiple-exponential function, and the corresponding decay time can be estimated using the following formula:

(3) y=A1∗exp(−x/t1)+A2∗exp(−x/t2)+A3∗exp(−x/t3)+y0

where A and y are the luminescence intensities at time 0 and x, respectively, and t is the decay lifetime [29].

(4) $$\tau_{\mathrm{ave}}=\frac{A1*t1^{2}+A2*t2^{2}+A3*t3^{2}}{A1*t1+A2*t2+A3*t3}$$

where τ indicates the average fluorescent lifetime. And the obtained lifetime values are 0.522, 0.556, 0.641, 0.807 and 1.12 ms for pH = 5, 7, 8, 10.5, and 13, respectively. The lattice distortion, particle size, and refractive index are the determining factors that affect the increase of the fluorescence lifetime according to previous report [30,31,32].

## 4. Conclusions

In this study, well dispersed and homogeneous Gd_2_O_2_S:Tb^3+^ phosphors with different morphologies (sphere-like, sheet-like, cuboid-like, flat square-like, rod-like) synthesized by changing pH value of reaction solution have been successfully achieved by a facile solvothermal method combining with a sulfide calcination process. We investigated the possible formation mechanisms of the phosphors with different morphologies and the morphology-dependent photoluminescence and cathodoluminescence properties. And the crystal growth model of the phosphors with different morphology was analyzed in detail. Phosphors with sphere-like morphology shows the best luminescence properties among these morphologies, and the emission color of the resulting phosphors was strongly affected by excitation source. Finally, Gd_2_O_2_S:Tb^3+^ phosphors with diverse morphologies, photoluminescence and cathodoluminescence properties provides more possibilities for systematically adjusting the green luminescence for a broad class of technological applications.

## Data Availability

The data presented in this study is available on request from the corresponding authors.

## References

[1] Thirumalai J., Chandramohan R., Vijayan T.A., Somasundaram R.M. (2011). Preparation of Highly Ordered Growth of Single-Crystalline Gd_2_O_2_S:Eu^3+^ Nanostructures. Mater. Res. Bull..

[2] Sang X., Lian J., Wu N. (2020). Synthesis Characterization and Formation Mechanism of Gd_2_O_2_S:Pr^3+^,Ce^3+^ Phosphors by Sealed Triple-Crucible Method. J. Asian. Ceram. Soc..

[3] Song Y., You H., Huang Y., Yang M., Zheng Y., Zhang L., Guo N. (2010). Highly Uniform and Monodisperse Gd_2_O_2_S:Ln^3+^ (Ln= Eu, Tb) Submicrospheres: Solvothermal Synthesis and Luminescence Properties. Inorg. Chem..

[4] Wang W., Kou H., Liu S., Shi Y., Li J., Li Y., Feng X., Pan Y., Guo J. (2015). Comparison of the Optical and Scintillation Properties of Gd_2_O_2_S:Pr, Ce Ceramics Fabricated by Hot Pressing and Pressureless Sintering. Opt. Mater..

[5] Katumo N., Ruiz Preciado L.A., Kumar V., Hernandez Sosa G., Richards B.S., Howard I.A. (2021). Anticounterfeiting Labels with Smartphone-Readable Dynamic Luminescent Patterns Based on Tailored Persistent Lifetimes in Gd_2_O_2_S:Eu^3+^ /Ti^4+^. Adv. Mater. Technol.-US.

[6] Lei L., Zhang S., Xia H., Tian Y., Zhang J., Xu S. (2017). Controlled Synthesis of Lanthanide-Doped Gd_2_O_2_S Nanocrystals with Novel Excitation-Dependent Multicolor Emissions. Nanoscale.

[7] Wang F., Yang B., Zhang J., Dai Y., Ma W. (2010). Highly Enhanced Luminescence of Tb^3+^-Activated Gadolinium Oxysulfide Phosphor by Doping with Zn^2+^ Ions. J. Lumin..

[8] Da Silva A.A., Cebim M.A., Davolos M.R. (2008). Excitation Mechanisms and Effects of Dopant Concentration in Gd2O2S:Tb3+ Phosphor. J. Lumin..

[9] Giakoumakis G.E., Nomicos C.D., Sandilos P.X. (1989). Absolute Efficiency of Gd_2_O_2_S:Tb Screens Under Fluoroscopic Conditions. Phys. Med. Biol..

[10] Huang X., Ding J., Liu X., Li X., Chen H., Hu D., Zhu D., Xie T., Zhou J., Jiang X. (2021). Fabrication of Gd_2_O_2_S:Tb Scintillation Ceramics From the Uniformly Doped Nanopowder. Opt. Mater..

[11] Yasuda R., Katagiri M., Matsubayashi M. (2012). Influence of Powder Particle Size and Scintillator Layer Thickness On the Performance of Gd_2_O_2_S:Tb Scintillators for Neutron Imaging. Nucl. Instrum. Methods Phys. Res. Sect. A Accel. Spectrometers Detect. Assoc. Equip..

[12] Zhang B., Zou H., Dai Y., Song Y., Zheng K., Zhou X., Sheng Y. (2016). Controlled Synthesis and Morphology Dependent Luminescence of Lu_2_O_2_S:Eu^3+^ Phosphors. RSC Adv..

[13] Wang F., Liu X. (2009). Recent Advances in the Chemistry of Lanthanide-Doped Upconversion Nanocrystals. Chem. Soc. Rev..

[14] Xie Y., Ma S., Wang Y., Xu M., Lu C., Xiao L., Deng S. (2018). Controlled Synthesis and Luminescence Properties of CaMoO_4_:Eu^3+^ Microcrystals. Opt. Mater..

[15] Jia K., Bi Z., Liu Y., Lyu Y. (2021). Electronic Structure, Morphology-Controlled Synthesis, and Luminescence Properties of YF_3_: Eu^3+^. J. Sol-Gel Sci. Technol..

[16] Zhang B., Zou H., Guan H., Dai Y., Song Y., Zhou X., Sheng Y. (2016). Lu_2_O_2_S:Tb^3+^, Eu^3+^Nanorods: Luminescence, Energy Transfer, and Multicolour Tuneable Emission. Crystengcomm.

[17] Xu G.X., Sang X.T., Lian J.B., Wu N.C., Zhang X. (2019). Solvothermal Synthesis and Luminescence Properties of Gd_2_O_2_S:RE^3+^ (RE^3+^= Eu^3+^/Tb^3+^) Hollow Sphere. Key. Eng. Mater..

[18] Liu Q., Pan H., Chen X., Li X., Liu X., Li W., Hu Z., Zhang X., Wu L., Li J. (2019). Gd_2_O_2_S:Tb Scintillation Ceramics Fabricated From High Sinterability Nanopowders Via Hydrogen Reduction. Opt. Mater..

[19] Liu F., Lian J., Wu N., He J., Zhang X., Liu F. (2018). One-Step Solvothermal Synthesis, a Worm-Shaped Morphology and Luminescence Properties of Green-Emitting Y_2_O_2_S:Tb^3+^ Nanophosphors. Opt. Laser. Technol..

[20] Sang X., Xu G., Lian J., Wu N., Zhang X., He J. (2018). A Template-Free Solvothermal Synthesis and Photoluminescence Properties of Multicolor Gd_2_O_2_S:X Tb^3+^, Y Eu^3+^ Hollow Spheres. Solid State Sci..

[21] Wan Y., Wang S., Luo W., Zhao L. (2012). Impact of Preparative pH on the Morphology and Photocatalytic Activity of BiVO_4_. Int. J. Photoenergy.

[22] Gómez-Barojas E., Sánchez-Mora E., Castillo-Abriz C., Flores-Rodríguez E., Silva-González R. (2013). Synthesis and Study of Optical and Photocatalytic Properties of Mn and Sm Doped ZnS Grown by Sol-Gel. J. Supercond. Nov. Magn..

[23] Jia G., Liu K., Zheng Y., Song Y., Yang M., You H. (2009). Highly Uniform Gd(OH)_3_ and Gd_2_O_3_:Eu^3+^ Nanotubes: Facile Synthesis and Luminescence Properties. J. Phys. Chem. C.

[24] Sahana M.B., Sudakar C., Dixit A., Thakur J.S., Naik R., Naik V.M. (2012). Quantum Confinement Effects and Band Gap Engineering of SnO_2_ Nanocrystals in a MgO Matrix. Acta Mater..

[25] Gholami T., Salavati-Niasari M. (2016). Effects of Copper:Aluminum Ratio in CuO/Al_2_O_3_ Nanocomposite: Electrochemical Hydrogen Storage Capacity, Band Gap and Morphology. Int. J. Hydrogen Energ..

[26] He C., Xia Z., Liu Q. (2015). Microwave Solid State Synthesis and Luminescence Properties of Green-Emitting Gd2O2S:Tb3+ Phosphor. Opt. Mater..

[27] Jeong G.J., Kang T.W., Park Y.J., Park Y.J., Lee Y., Bae B., Kim S.W. (2021). Development of a Cyan Blue-Emitting Ba_3_La_2_(BO_3_)_4_:Ce^3+^, Tb^3+^ Phosphor for Use in Dental Glazing Materials: Color Tunable Emission and Energy Transfer. RSC Adv..

[28] Hernandez-Adame L., Palestino G., Meza O., Hernandez-Adame P.L., Vega-Carrillo H.R., Sarhid I. (2018). Effect of Tb^3+^ Concentration in the Visible Emission of Terbium-Doped Gadolinium Oxysulfide Microspheres. Solid State Sci..

[29] Chen J., Song Y., Li D., Ma Q., Dong X., Yu W., Wang X., Yang Y., Wang J., Liu G. (2019). Investigating Efficient Energy Transfer in Novel Strategy-Obtained Gd_2_O_2_S:Dy^3+^, Eu^3+^ Nanofibers Endowed with White Emitting and Magnetic Dual-Functionality. J. Lumin..

[30] Birkel A., Denault K.A., George N.C., Doll C.E., Héry B., Mikhailovsky A.A., Birkel C.S., Hong B., Seshadri R. (2012). Rapid Microwave Preparation of Highly Efficient Ce^3+^-Substituted Garnet Phosphors for Solid State White Lighting. Chem. Mater..

[31] Zhu H., Lin C.C., Luo W., Shu S., Liu Z., Liu Y., Kong J., Ma E., Cao Y., Liu R. (2014). Highly Efficient Non-Rare-Earth Red Emitting Phosphor for Warm White Light-Emitting Diodes. Nat. Commun..

[32] Singh S., Singh D. (2021). Structural and Optical Properties of Green Emitting Y_2_SiO_5_:Tb^3+^ and Gd_2_SiO_5_:Tb^3+^ Nanoparticles for Modern Lighting Applications. Rare Metals.

